# How Experimental Adjustments and Optimizations led to Success in obtaining Enzyme-Ligand Crystal Structures of Zymoseptoria tritici ACCase Carboxyltransferase domain

**DOI:** 10.1063/4.0001011

**Published:** 2025-10-27

**Authors:** Timothy J. Rydel, Rachappa Balkunde, Timothy Boyle, Rong Ma, Paramita Deria, Michael Crawford, Virginie Lempereur, Tommi White

**Affiliations:** 1 Bayer Research and Development Services LLC, Chesterfield, MO, USA; 2 Bayer S.A.S., La Dargoire, FRANCE, France

## Abstract

Zymoseptoria tritici (Z. tritici) is a wheat pathogen that causes septoria leaf blotch, and it is one of the most economically damaging diseases of wheat. The Structural Biology Workflow, which is in the Protein Sciences Hub of the Molecular & Cellular Sciences Capability Center, partnered with colleagues in Target Key to pursue X-ray crystal structures of the Acetyl-coenzyme A carboxylase (ACCase) Carboxyltransferase domain. The aim of this collaboration was to determine protein structures with company fungicidal molecules bound. It was a challenging project, but we ultimately were able to determine four unique crystal structures of the Z.t. ACCase_CT domain: that of the enzyme alone and of three unique protein-ligand complexes using Bayer inhibitors. This talk will highlight how making experimental adjustments/optimizations throughout the project led to success, and what we learned about these protein complexes in the process.

